# Fluorescent Aptasensor and Colorimetric Aptablot for p-tau231 Detection: Toward Early Diagnosis of Alzheimer’s Disease

**DOI:** 10.3390/biomedicines10010093

**Published:** 2022-01-01

**Authors:** Le Minh Tu Phan, Sungbo Cho

**Affiliations:** 1 Department of Electronic Engineering, Gachon University, Seongnam-si 13120, Korea; 2 School of Medicine and Pharmacy, The University of Danang, Danang 550000, Vietnam; 3 Department of Health Sciences and Technology, GAIHST, Gachon University, Incheon 21999, Korea

**Keywords:** aptamer, nitrogen-doped carbon dot, gold nanoparticle, copper-enhanced gold, enhanced colorimetric signal

## Abstract

The pathology of Alzheimer’s disease (AD), the most common cause of dementia, is considered to be mainly driven by two major hallmarks (tau and amyloid beta). It is highly desirable to develop an affordable medicinal diagnostic that can be utilized worldwide for the early diagnosis of AD. Hence, p-tau231 was selected as a specific target, which appears both in AD serum and cerebrospinal fluid, for the development of a sensing platform for the diagnosis of AD. To the best of our knowledge, these are the first aptamer-mediated biosensors that rely on sensitive fluorescent and colorimetric aptasensors for the rapid monitoring of p-tau231. The nitrogen-doped carbon dot-based turn-on fluorescent aptasensor could rapidly analyze p-tau231 down to 3.64 ng/mL within 40 min, and the colorimetric Cu-enhanced-Au aptablot displayed high sensitivity at 4.71 pg/mL through a digital camera, with visibility to the naked eye down to 8 ng/mL p-tau231 within 140 min. Owing to their advantages, which include affordability, rapidity, high sensitivity, and dependence on complicated instruments, these aptamer-based biosensors offer significant potential for the early diagnosis of AD worldwide.

## 1. Introduction

Alzheimer’s disease (AD) is the most common form of neurodegenerative dementia that severely endangers the elderly, both physically and mentally. The disease is characterized by the presence of extracellular senile plaques and intracellular neurofibrillary tangles in the brain; the former is due to the aggregation of amyloid β peptide (Aβ), while the latter is formed by the polymerization of phosphorylated tau protein [1,2,3]. Among various molecules used as the biomarker for AD diagnosis, phosphorylated tau (p-tau) is a specific pathological biomarker that represents the early onset of AD progression [4,5]. Several p-tau isotypes have been investigated for the diagnosis of AD pathogenesis in the early stages, such as p-tau181, p-tau231, p-tau217, and p-tau231. Among them, p-tau231 has emerged as a highly specific pathological biomarker of AD that remains normal in other dementia diseases [6,7]. p-tau231 is also superior to p-tau181 or p-tau217 for distinguishing AD patients from healthy people, and those with other neurodegenerative disorders. The detection of p-tau231 would also be beneficial in reflecting the early abnormalities in Aβ. However, to date, there is no rapid and specific biosensor for the detection of p-tau231, which would be beneficial for AD diagnosis. Therefore, it is essential to develop a straightforward biosensor that allows accurate diagnosis of AD and early intervention based on p-tau231 detection.

The nanobiosensor development for AD diagnosis has been well investigated during the last decade, with a variety of techniques, such as ELISA, colorimetric assay, and electrochemical assays [8,9,10]. However, limitations to these existing techniques towards the early and precise diagnosis of AD have remained, which include the high cost and complex instrument setup requirement. Compared with conventional antibodies, the employment of aptamers as a biorecognition element offers distinctive advantages, including the facile chemical synthesis, high stability, high reproducibility, and good sensitivity [11,12]. The integration of such aptamer recognition elements into biosensor platforms can result in a satisfactory strategy for broad applications. Hence, various types of colorimetric and fluorescent probes have been investigated for the fabrication of colorimetric and fluorescent aptasensors, respectively. Among several potential nanomaterials, carbon dots (CD) have attracted tremendous attention, owing to their high photostability, excellent biocompatibility, and easy preparation methods [13,14].

**Figure 1:**
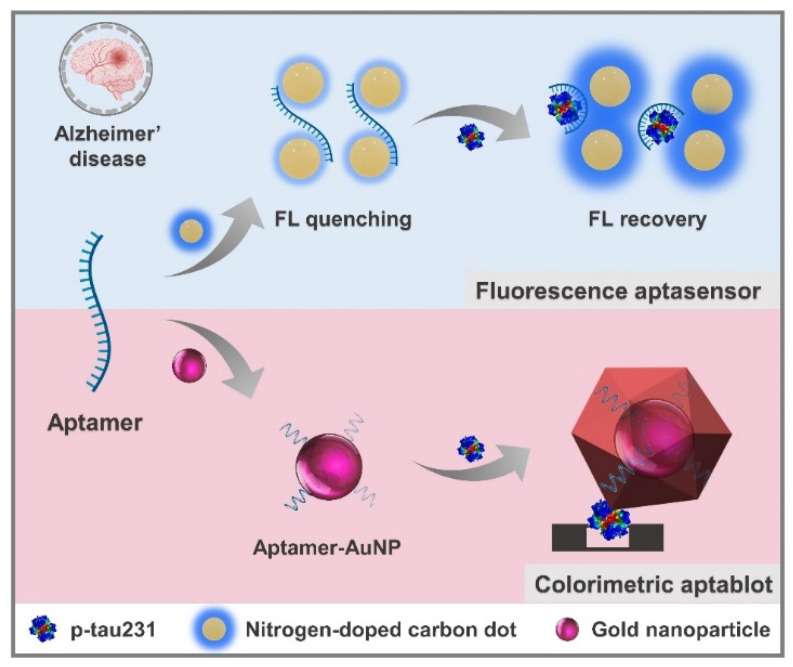
Representation of aptamer-mediated diagnostics toward early and accurate diagnosis of Alzheimer’s disease through the rapid and sensitive detection of p-tau231 using turn-on fluorescent NCDs-based aptasensor and naked-eye colorimetric Cu-enhanced-Au aptablot.

In the present work, we have developed two promising aptasensors for the detection of p-tau231 as AD biomarkers, including a fluorescent aptasensor and a colorimetric aptablot, for the detection of p-tau231, using fluorescent nitrogen-doped CDs (NCDs), gold nanoparticle (AuNPs), and specific aptamer of p-tau231. Figure 1 demonstrates the primary principles of p-tau231 detection through the fluorescent aptasensor and the colorimetric aptablot that are of potential use for the early diagnosis of AD. By monitoring the change in fluorescent or colorimetric intensity, the level of target molecules can be evaluated due to the signal dependence on the concentration of the target analyte. For the rapid detection of p-tau231, a label-free fluorescent NCD-based aptasensor has been developed, which includes two steps of fluorescence quenching and recovery. The aptamer is assembled on the surface of NCDs, causing fluorescence quenching. In the presence of p-tau231, the aptamer specifically binds to p-tau231 and dissociates from the NCDs, leading to the recovery of NCD fluorescence, which could be used for the quantification of p-tau231. For sensitive naked-eye detection of p-tau231, a colorimetric Cu-enhanced-Au aptablot has been exploited to improve the colorimetric intensity through the enlargement of the size of AuNPs and the transformation of the particle shape, becoming visible to the naked eye for the detection of p-tau231. These aptasensing approaches were successfully developed to selectively detect p-tau231 in serum, exhibiting significant potential in the early diagnosis of AD. With their superior properties of cost-effectiveness, simple and rapid process, and high sensitivity, these diagnostic tools exhibit excellent ability for the sensitive detection of p-tau231, thus conferring promising potential for application in the early diagnosis of AD.

## 2. Methods

### 2.1. Materials

All reagents were of analytical grade and were used for the experiment without further purification. Deionized (DI) water at 18.2 MΩ cm was purified using a Milli-Q system (Purescience, Gyeonggi, Korea). Citric acid monohydrate (C_6_H_8_O_7_·H_2_O) and sodium citrate dihydrate (C_6_H_5_Na_3_O_7_·2H_2_O) were purchased from OCI Company (Seoul, Korea). Polyethylenimine (PEI1800, branched average molecular weight 1800) and sodium ascorbate were obtained from Alfa Aesar (Ward Hill, MA, USA). Tetrachloroauric(III) acid (HAuCl_4_·3H_2_O) was purchased from Sigma–Aldrich (Seoul, Korea). Nitrocellulose membrane was purchased from Nupore, India. Human serum albumin (HSA) was also received from Sigma–Aldrich (Seoul, Korea). Phosphate buffer saline (PBS, pH 7.4) was purchased from Tech-Innovation (Seoul, Korea). p-tau231, amyloid beta 1–42 peptide (Aβ42), C-reactive protein (CRP), tumor necrosis factor α (TNF-α), and insulin were obtained from Abcam (Seoul, Korea). Amyloid beta 1–42 aggregates containing Aβ42 oligomer were obtained from Anaspec (Fremont, CA, USA). Thiol-modified AFB1 aptamer (5′-HS-(CH_2_)_6_-CAGCACCGTCAACTGAATGGGGAGAGTGGTGGGGCGGGGGCCGGATCCGTGATGCGATGGAGATGT-3′) was synthesized by Genotech Corp. (Yuseong, Korea), according to identification from the literature [15].

### 2.2. Synthesis and Characterization of NCDs

Before use, all glassware and autoclave reactors used for the experiments were thoroughly washed with deionized (DI) water, and air-dried. NCDs were successfully synthesized using a hydrothermal method. Citric acid monohydrate (4.0 g, 19 mmol) and PEI1800 (1.03 g, 1.25 mmol) were dissolved in 30 mL DI water, and vigorously stirred for 30 min at room temperature (RT). The mixture solution was placed in a Teflon-lined autoclave reactor, and heated at 190 °C for 1 h. A short reaction time (1 h) was chosen to fabricate the NCDs to produce NCDs with high quantum yield [16]. NCDs were hydrothermally formed by combination between citric acid as the carbon source, and PEI1800 as the nitrogen source. During hydrothermal synthesis, pyridine-type fluorophore intermediates are formed by dehydration, and remain on the particle surface [17]. After hydrothermal reaction, a yellow solution containing NCDs was obtained after cooling to RT, followed by filtering through 0.22 µm micron filter to remove the large particles. Absolute ethanol was then added to the resultant solution, which was vortexed for 30 s, and washed by centrifugation (8000 rpm for 1 min at 25 °C) to remove excess citric acid and PEI1800, due to their high ethanol solubility. The pellets were collected, dispersed in DI water, and stored at 4 °C for further characterization and usage.

The physical properties of the as-prepared NCDs were obtained through characterization. The size of NCDs was obtained by high-resolution transmission electron microscopy (TEM-FEI Tecnai, Waltham, MA, USA). The structures of the different components of the NCDs were analyzed through X-ray photoelectron spectroscopy (XPS) spectra using X-ray photoelectron spectrometry (K-alpha+, ThermoFisher Scientific, Waltham, MA, USA). X-ray diffraction (XRD) data were measured on SmartLab^®^ X-ray diffractometer (Rigaku, Japan). UV-Vis and fluorescent spectra of NCDs were acquired by multi-mode microplate reader (96-well microplate, BioTek Synergy H1, Winooski, VT, USA).

### 2.3. Turn-On Fluorescent NCDs-Based Aptasensor to Detect p-tau231

The p-tau231 aptamer solution (1.5 µM, diluted in PBS buffer) was incubated at 90 °C for 5 min, and gradually cooled to RT for 15 min. One hundred microliters of p-tau231 aptamer solution was mixed with NCDs in 1.5 mL microcentrifuge tube to quench the fluorescence of NCDs. The mixture solution was thoroughly pipetted, and equilibrated for different incubation times (0–50) min at 25 and 37 °C. The final concentration of Mg^2+^ (1 mM) and different concentrations of p-tau231 contained in PBS (180 µL) were introduced to the NCDs/aptamer complex solution, and incubated for different incubation time at 37 °C. Finally, the fluorescence intensities of the reaction mixture were continuously measured under 365 nm excitation. The fluorescence recovery after the addition of p-tau231 was evaluated by calculating the intensity ratio (F’-F)/F, where F is the fluorescence emission intensity after fluorescence quenching by aptamer, and F’ is the recovery fluorescence intensity after adding p-tau231. To monitor the interference effects of different biomarkers that appear in human serum, such as human serum albumin (HSA), amyloid beta 42 monomer (Ab42), amyloid beta 42 oligomer (AbO42), C-reactive protein (CRP), tumor necrosis factor α (TNF-α), and insulin; 2.5-fold concentration of these biomarkers was used to measure the interference effect to turn-on the fluorescent aptasensor for p-tau231.

### 2.4. Colorimetric Cu-Enhanced-Au Aptablotting to Detect p-tau231

Gold nanoparticles were synthesized by the reduction of sodium citrate, following the literature [18]. The aptamer of p-tau231 was functionalized onto the surface of AuNPs by addition into AuNPs solution and incubation for 4 h after heating at 90 °C and cooling; then, the solution was washed 2 times by centrifugation. Multiple reaction chambers were created in nitrocellulose membrane using wax printing and heating at 95 °C, following the literature [19]. For different concentrations of p-tau231 of (0–1000 ng/mL), 2 µL was dropped onto the different reaction chambers, dried for 10 min, and followed by a blocking step with bovine serum albumin for 45 min. After washing, the reaction chambers were immersed in the aptamer-functionalized AuNPs solution for 60 min, following by immersion in the copper enhancing solution (CuCl_2_, PEI1800, and sodium ascorbate) to improve the color intensity [20]. These color intensities were analyzed by the naked eye or by digital camera with the support of ImageJ 1.53e software.

## 3. Results and Discussions

### 3.1. Physical Characterization of NCDs

NCDs were successfully synthesized using citric acid and PEI1800 as carbon source and nitrogen source, respectively, through a one-step hydrothermal method. The morphology and size of NCDs confirmed by TEM show the spherical shape of the as-synthesized NCDs with estimated size of 3.5–4.5 nm, and the zeta potential of +27.0 mV indicates the high dispersibility and colloidal stability of the NCDs (Figure S1 of the Supporting Information (SI)). During the hydrothermal reaction, amides and pyridine-type fluorophores were formed by dehydration, followed by carbonization within 1 h, and remained on the surface of NCDs covalently [17]. Therefore, different functional groups, including hydroxyl, carboxyl, and amino groups, appeared on the particle surface. XRD and XPS characterizations were performed to validate the composition and structure of the NCDs. The diffraction peak at 2θ = 19.58° in the XRD pattern suggests the formation of amorphous carbon (Figure S2 of the SI). XPS survey showed the composition of C (63.6%), O (24.8%), and N (11.4%) with the element states of O 1s, N 1s, and C 1s corresponding to the strong binding energy peaks at 530.08, 399.08, and 285.08 eV, respectively. In the high-resolution spectrum of C 1s, N 1s, and O 1s, the three peaks at 284.5, 285.9, and 284.7 eV are attributed to the C=O/C–O, C–N, and C–C groups, respectively; the two peaks at 400.7 and 399.1 eV are attributed to the N–H and C–N–C groups, respectively; and the two peaks at 530.4 and 532.1 eV are attributed to the C=O and C–O/N–O groups, respectively (Figure S3 of the SI). These results thus indicate that the NCDs have the three elements of C, N, and O, with positive charge for further application.

**Figure 2:**
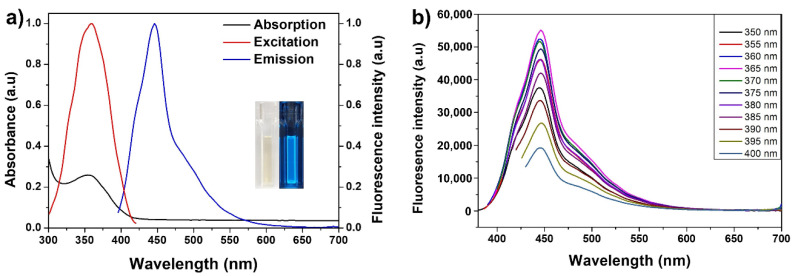
Optical properties of NCDs. (**a**) UV–Vis absorption and fluorescence spectra demonstrate the maximum absorption wavelength located at 365 nm, and the excitation and emission wavelength at 365 and 445 nm, respectively. Inset: photographs of NCDs solution under visible light (left) and 365 nm UV irradiation. (**b**) The effect of different excitation wavelengths in the range 350–400 nm on the fluorescence emission indicates that the maximum fluorescence intensity was recorded at a 365 nm excitation wavelength.

Due to the fluorescence-based sensing application of NCDs, it was crucial to investigate the optical properties of the NCDs for further application. UV–Vis and fluorescence spectra were performed to evaluate their detailed properties. The NCDs exhibit a pale yellow color in daylight, which observed the characteristic absorption peak at 357 nm that originated from the n–π transition of C=O bonds [21]. Further, NCDs displayed strong blue fluorescence under UV irradiation at 365 nm. The fluorescence emission peak was obtained at 445 nm when excited at 365 nm, indicating the strong dependence of the fluorescence excitation spectrum on the observation wavelength (Figure 2a). Different excitation wavelengths from 350 to 400 nm were analyzed to monitor the optimal fluorescence condition through the excitation-dependent behavior of NCDs. Figure 2b shows that the fluorescence emission intensity differs, but the emission peak does not shift under different excitation variation, exhibiting the maximum emission wavelength at 445 nm under excitation wavelength at 365 nm. Additionally, the long-term stability of the sensing platform is an important criterion for use in sensing applications. Hence, the fluorescence stability of NCDs was recorded after 3 months storage at 4 °C in a dark environment, which showed no significant change in fluorescence emission intensity (11.2% intensity reduction) (Figure S4 of the SI), suggesting that NCDs probes could be stored for a long time for further applications, without elimination of the fluorescence properties.

### 3.2. Turn-On Fluorescent NCDs-Based Aptasensor for the Rapid Detection of p-tau231

**Figure 3:**
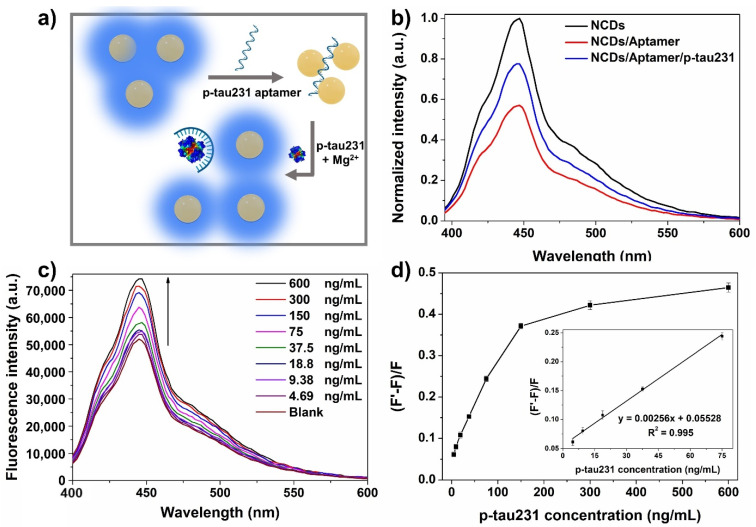
Investigation into the sensing performance of turn-on fluorescent NCDs-based aptasensor for p-tau231. (**a**) Schematic of the fluorescent aptasensor mechanism including two steps: fluorescence quenching upon the electrostatic adsorption of positively charged NCDs on negatively charged aptamers, followed by the release of free NCDs to restore fluorescence after p-tau231 introduction, due to the stronger binding affinity of the aptamer toward p-tau231 than NCDs. (**b**) Fluorescence spectra of the initial NCDs, NCDs + aptamer, and NCDs + aptamer + p-tau231 indicate the significant fluorescence recovery after the addition of p-tau231 into the NCDs/aptamer complex. (**c**) Fluorescence recovery spectra of NCDs with increasing p-tau231 concentration of 0–600 ng/mL. (**d**) Relative fluorescence intensity of aptasensor for the detection of p-tau231 corresponding to different p-tau231 concentrations. Inset: linear regression between p-tau231 concentrations of 0–75 ng/mL and fluorescence recovery efficiency ((F’−F)/F).

p-tau231 is considered as a promising biomarker of the emerging AD pathology because p-tau231 is neuropathologically increased earlier than p-tau181, not only prior to the positive threshold of amyloid beta, but in response to early brain tau deposition [6,7,22]. Therefore, monitoring the presence of plasma p-tau231 could be essential for the early diagnosis of AD. We developed the fluorescent aptasensor for p-tau231 using the aptamer of p-tau231, which was identified by Teng’s group [15]. Figure S5 of the SI shows the secondary structure of p-tau231 aptamer that was predicted using the web server for RNA secondary structure prediction (https://rna.urmc.rochester.edu/RNAstructureWeb, accessed on 8 March 2021). Figure 3a shows the principle of the turn-on fluorescent aptasensor for p-tau231 detection. The p-tau231 aptamer used in the aptasensor is a single-stranded oligonucleotide that possesses abundant negative charges along the structure of the aptamer due to the presence of phosphate backbone [23,24]. Upon the addition of p-tau231 aptamer into NCDs solution, positively charged NCDs could absorb onto the aptamer through electrostatic interaction, leading to the fluorescence quenching effect due to fluorescence resonance energy transfer. Owing to the high affinity and specificity of the aptamer to the target, once p-tau231 is introduced into the complex NCDs/aptamer solution along with Mg^2+^ ions, Mg^2+^ ions (final concentration of 1 mM) could accelerate the saturated folding transition of aptamers into the specific shape that selectively bind to their targets [25,26], leading to the release of NCDs that could recover the fluorescence intensity. The proof of principle is supported by the fluorescence performance of this aptasensor, as shown in Figure 3b. The fluorescence emission intensity of the NCDs solution under 365 nm UV irradiation sharply reduced after the addition of aptamer, confirming the quenching effect of aptamer towards the NCDs. However, the fluorescence recovery was observed after the introduction of p-tau231 biomarker through the significant increase in fluorescence intensity. These results confirm the potential of this aptasensor for the detection of p-tau231 in early AD diagnosis.

To achieve the optimal performance of the aptasensor, the quenching effect of the aptamer towards fluorescent NCDs was monitored by optimizing the incubation temperature and incubation time. Figure S6 of the SI shows the dependence of fluorescence quenching on the temperature (25 and 37 °C) and incubation time (10–50 min). The fluorescence intensity gradually decreased with increasing the incubation time at both 25 and 37 °C; however, the quenching effect occurred faster at 37 °C as the physiological temperature, with almost maximum quenching at 30 min. Therefore, 30 min of incubation at physiological temperature was chosen as the optimal quenching condition of aptamer toward NCDs. The next reaction was then performed at 37 °C to keep the remarkable biological activity of aptamer and p-tau231 [15]. At 37 °C, the fluorescence recovery effect was further investigated with different time intervals from 5–50 min after p-tau231 addition into the NCDs/aptamer complex solution (Figure S7 of the SI). The first period of incubation time shows that the fluorescence intensity decreased due to the light fluorescence quenching effect of the high concentration of Mg^2+^ ions that were added along with p-tau231, according to the above interference study of ferrous ions detection. However, after 15 min, the fluorescence recovery was gradually observed, and reached equilibrium recovery within 40 min incubation time. The 40 min incubation time after the addition of p-tau231 was chosen for further experiments. Figure 3c shows the fluorescence spectra of NCDs/aptamer/p-tau231 at different concentrations of p-tau231 in the ranged of 0 to 600 ng/mL. The fluorescence intensity of the NCDs/aptamer/p-tau231 solution progressively increased with the increased concentration of p-tau231. The fluorescence recovery effect was calculated according to the term (F’−F)/F, where F’ and F are the peak intensity of fluorescence emission at 445 nm after and before adding p-tau231, respectively. There is a correlation between the recovery effect and the concentration of p-tau231, with a good linear relationship (R^2^ = 0.995) in the range 0–75 ng/mL (Figure 3d). The limit of detection was found to be 3.64 ng/mL by calculation based on the 3σ/slope. In addition, specificity in the sensing platform is an important quality to evaluate the potential sensing performance. Hence, the specific ability of this fluorescent aptasensor was validated using other interferences in human serum (Figure S8 of the SI), including amyloid beta 42 (Ab42), amyloid beta oligomer 42 (AbO42), C-reactive protein (CRP), tumor necrosis factor (TNF), insulin, and human serum albumin (HSA) spiked in diluted serum samples, with a p-tau231 concentration of 200 ng/mL and other interferences at a concentration of 400 ng/mL. The fluorescence intensity was restored significantly in the mixture of other interferences with p-tau231, whereas it remained same fluorescence quenching in the mixture without p-tau231, indicating the specificity of this platform.

### 3.3. Colorimetric Cu-Enhanced-Au Aptablotting for the Sensitive Detection of p-tau231

**Figure 4:**
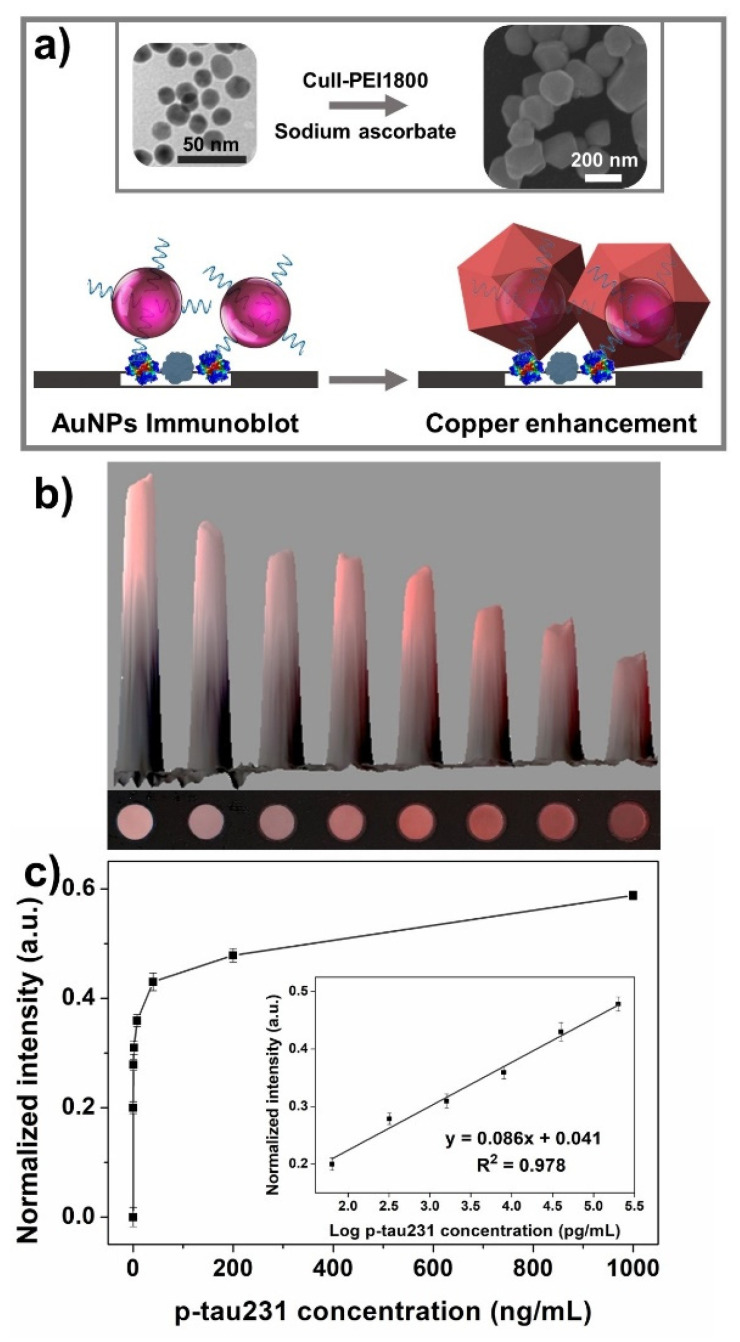
Investigation into the sensing performance of colorimetric Cu-enhanced-Au aptablotting for p-tau231. (**a**) Schematic of the colorimetric aptasensor mechanism. After direct immunoblotting using aptamer-functionalized AuNPs, the copper complex reduced by sodium ascorbate was absorbed onto AuNPs, which stimulated the enhancement of color intensity through particle size enlargement and shape transformance. Inset shows the TEM and SEM images of AuNPs and AuNPs-Cu before and after the addition of AuNPs into copper-enhanced solution, respectively. (**b**) The smartphone camera photographs and 3D histogram of reaction apta-based immunoblot with increasing concentrations of p-tau231 of 0, 0.064, 0.32, 1.6, 8, 40, 200, and 1000 ng/mL, clearly visible to the naked eye down to 8 ng/mL. (**c**) Color intensities of different concentration of p-tau231 of 0–1000 ng/mL. Inset is the calibration curve for the quantitative monitoring of p-tau231 concentration.

Naked-eye and sensitive detection approaches are highly necessary to detect the specific biomarkers of AD for early AD diagnosis. Figure 4a shows the copper-enhanced-gold dot-blot aptasensor that was used to measure the concentration of p-tau231 instead of the fluorescent aptasensor. The principle of this platform is based on the signal amplification of the direct dot-blot assay using a copper-enhancing method. The CuII-PEI complex was reduced, and exhibited the ability to absorb onto the surface of AuNPs, leading to the enlargement of nanoparticle size from about 16 nm for AuNPs to ~310 nm for AuNPs–Cu, as well as the transformation of particle shape from spherical to polygonal (inset of Figure 4a), suggesting the effective capability for signal amplification [20]. The color intensity analyzed through photographing the reaction chambers exhibited a good linear relationship (R^2^ = 0.978) in the p-tau231 concentration range of 0–1000 ng/mL (Figure 4c). After the total 140 min process, the effective observation of different colors could quantitatively analyze p-tau231 with the naked eye down to 8 ng/mL, as shown in Figure 4b, in the quantitative measure of p-tau231 through a digital camera, the LOD of 4.71 pg/mL was obtained. These results confirmed the ability of this colorimetric aptasensor for the sensitive detection of p-tau231 without the need for complicated equipment or technical professionals, suggesting it has promising potential for the monitoring of AD worldwide.

### 3.4. Feasibility of These Aptamer-Mediated Nanoplatforms for Accurate Diagnosis of Alzheimer’s Disease

Among several p-tau biomarkers for AD, p-tau231 is a superior biomarker in distinguishing AD patients from healthy people. Therefore, the use of tau as a clinical biomarker of AD means that their determinations could improve the accuracy of diagnosis and the monitoring of therapeutic response [27]. Plasma p-tau231 not only displays the ability to identify AD patients, and to differentiate them from amyloid-β-negative cognitively unimpaired older adults, but also to distinguish AD patients with neurodegenerative disorders [6]. The diagnostic cutoff value of p-tau231 in cerebrospinal fluid is about 10.1 ng/mL, which was able to differentiate between AD patients and non-AD patients [28]. The as-prepared turn-on fluorescent NCD-based aptasensor and signal-amplified aptablotting are feasible to detect p-tau231 rapidly and sensitively in any samples for the early and accurate diagnosis of AD, depending on the laboratory facilities and equipment, supporting their potential application within the clinical setting. These highly sensitive colorimetric and fluorescent aptasensors, which do not require a time-consuming process, could be always available for medical staff to accurately diagnose AD, as well as to validate the possible prognostic pathology of AD, efficiently specifying targeting tau therapy [29].

## 4. Conclusions

This study demonstrated efficient aptamer-based biosensors for the detection of p-tau231 as a turn-on fluorescent NCDs-based aptasensor and colorimetric Cu-enhanced-Au aptablotting. The straightforward fluorescent and colorimetric aptasensors for AD biomarker were successfully developed using a specific aptamer toward p-tau231, owing to the versatile turn on–off fluorescence of NCDs with target analytes, as well as the colorimetric signal amplification via a copper enhancement method, rapidly detecting p-tau231 through the fluorescent NCDs-based aptasensor (40 min, LOD 3.64 ng/mL) and the colorimetric AuNPs-based aptablot (140 min, LOD 4.71 pg/mL), respectively. Although these approaches displayed high specificity towards other AD biomarkers, further characterization is required to assess the specificity of these approaches towards other phosphoepitopes. These proposed approaches exhibit many obvious advantages that include multifunctionality, low cost, simple and rapid sensing process, high sensitivity and specificity, and independence from complicated instruments and technical professionals. These aptamer-based biosensors for p-tau231 might significantly pave the way for diagnostic biosensors towards the early diagnosis of AD worldwide.

## Data Availability

The dataset generated and analyzed in this study is not publicly available, but may be obtained from the corresponding author upon reasonable request.

## Supplementary Materials

The following supporting information can be downloaded at: https://www.mdpi.com/article/10.3390/biomedicines10010093/s1.
