# Supplementary material for: Fluorescent Aptasensor and Colorimetric Aptablot for p-tau231 Detection: Toward Early Diagnosis of Alzheimer’s Disease
Source: Biomedicines. 2022 Jan 1;10(1):93. doi: 10.3390/biomedicines10010093 (PMC8772959; doi:10.3390/biomedicines10010093)
Supplement: Supplementary file 1 [file biomedicines-10-00093-s001.zip › biomedicines-1521048-supplementary.pdf]

Supplementary Information for

Fluorescent Aptasensor and Colorimetric Aptablot for p-tau231 Detection: Toward Early Diagnosis of Alzheimer's Disease

Le Minh Tu Phan[a,b] *, and Sungbo Cho[a, c, *]

[a]*Department of Electronic Engineering, Gachon University, Seongnam-si, Gyeonggi-do, 13120, Republic of Korea*

[b]*School of Medicine and Pharmacy, The University of Danang, Danang 550000, Vietnam*

[c]*Department of Health Sciences and Technology, GAIHST, Gachon University, Incheon, 21999, Republic of Korea*

** Corresponding authors:* plmtu@smp.udn.vn (Le Minh Tu Phan); sbcho@gachon.ac.kr (Sungbo Cho)

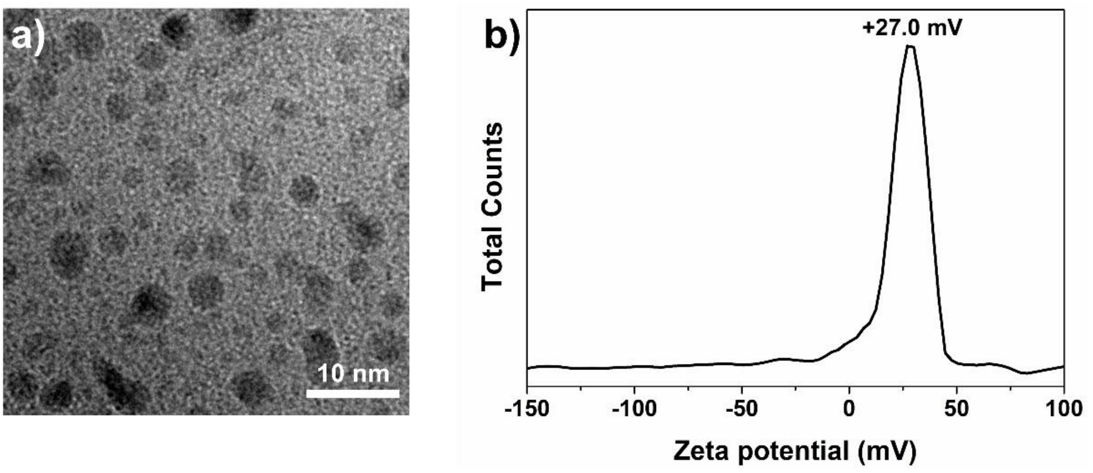

Figure S1. a) TEM image of NCDs. b) Zeta potential distribution of NCDs.

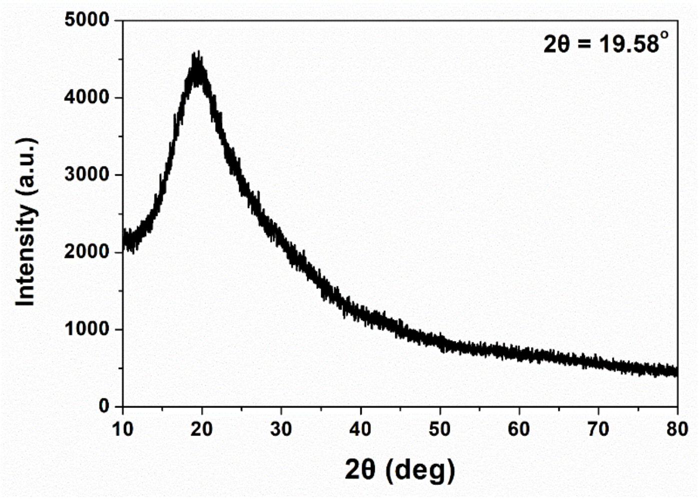

Figure S2. XRD pattern of NCDs.

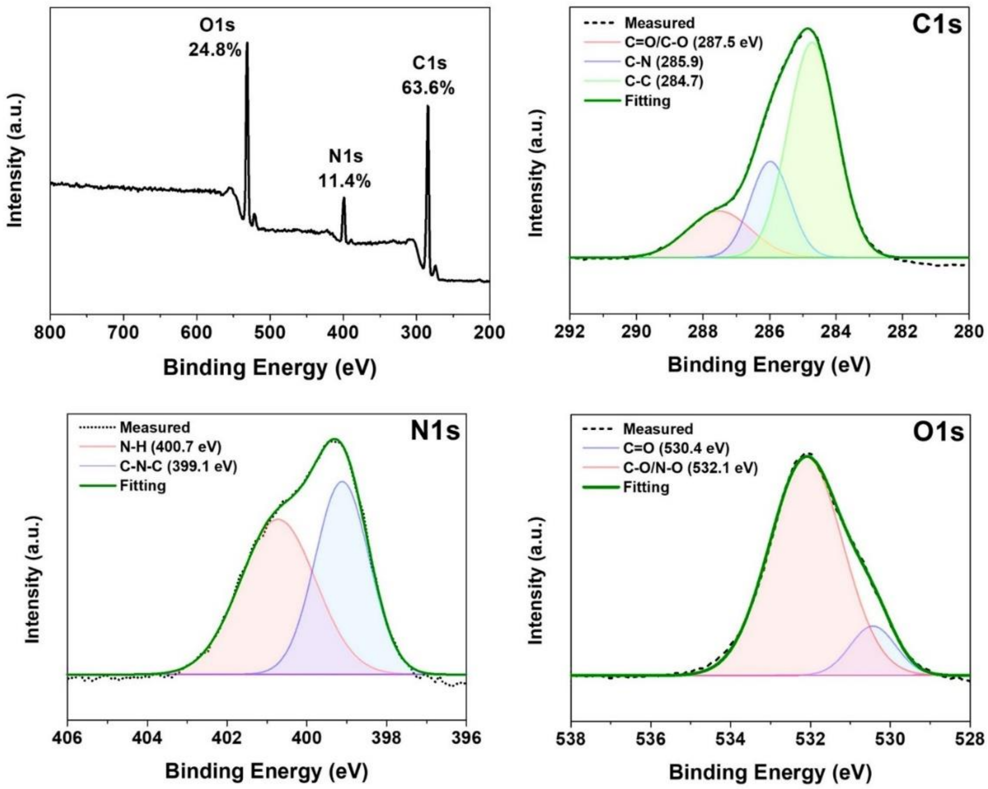

Figure S3. XPS survey scan of NCDs including full scan spectrum and high-resolution survey scan of C1s, N1s, and O1s.

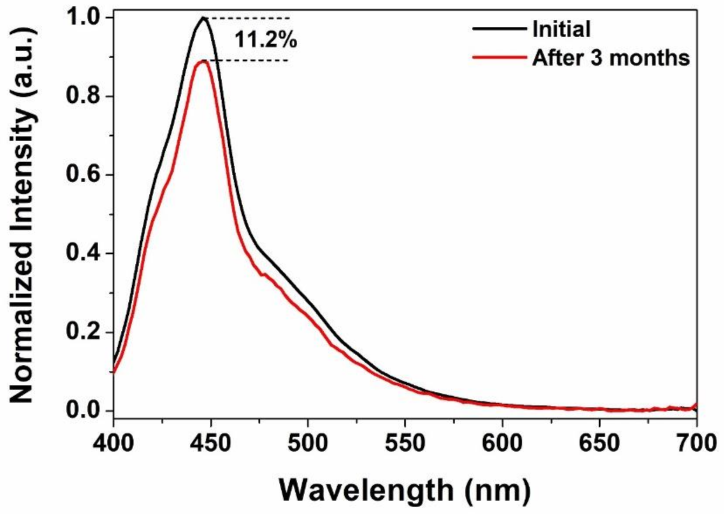

Figure S4. Fluorescence stability of NCDs after storing for 3 months at 4 °C in the dark environment.

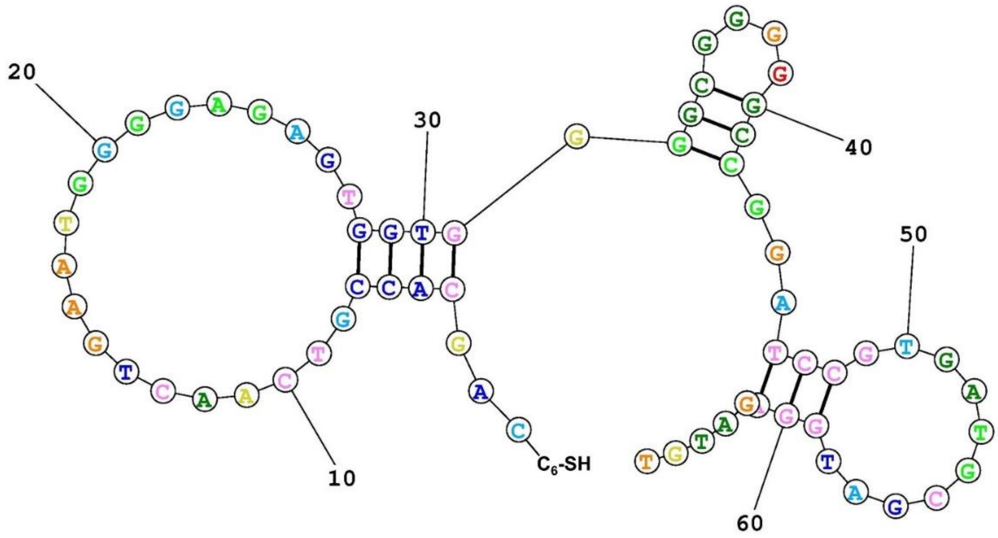

Figure S5. Secondary structure prediction of p-tau231 aptamer sequence with lowest free energy using RNAstructure software.

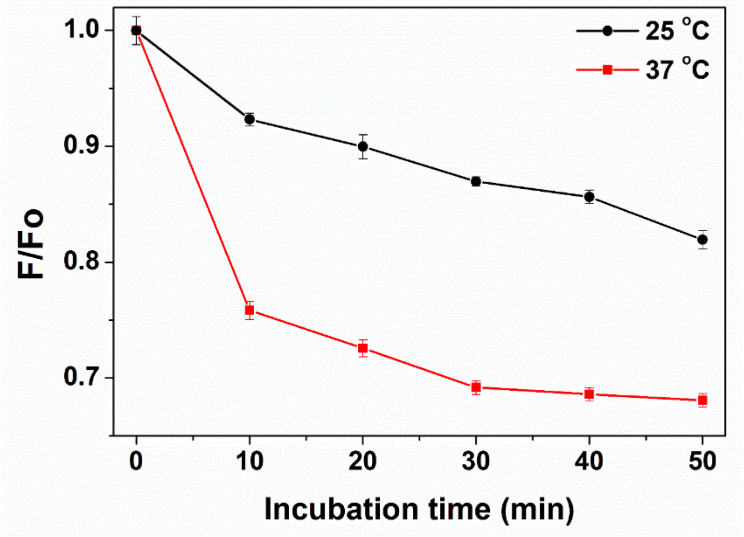

Figure S6. Influence of incubation temperature and incubation time on the quenching effect of p-tau231 aptamer toward NCDs.

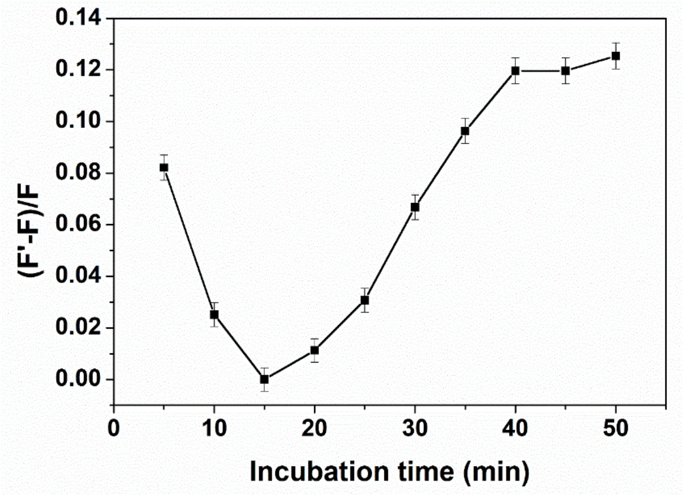

Figure S7. Effect of incubation time after p-tau231 addition on recovery efficiency of fluorescent NCDs.

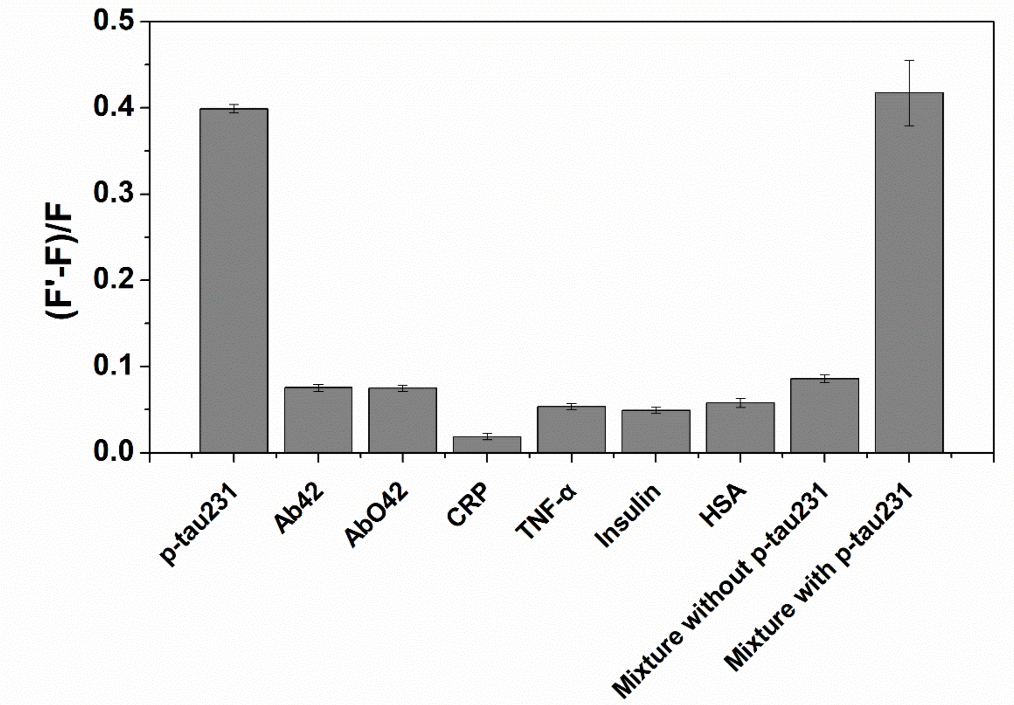

Figure S8. Specificity validation of turn-on fluorescent aptasensor for p-tau231 peptide by examining effect of interferences in spiked serum samples including p-tau181 peptide, amyloid

beta 42 monomer (Ab42), amyloid beta 42 oligomer (AbO42), C-Reactive protein (CRP), tumor necrosis factor (TNF), insulin, human serum albumin (HSA).
